# Neurophysiological mechanisms of interval timing dissociate inattentive and combined ADHD subtypes

**DOI:** 10.1038/s41598-018-20484-0

**Published:** 2018-02-01

**Authors:** Annet Bluschke, Jacqueline Schuster, Veit Roessner, Christian Beste

**Affiliations:** 1 Cognitive Neurophysiology, Department of Child and Adolescent Psychiatry, Faculty of Medicine of the TU Dresden, Dresden, Germany; 2 Experimental Neurobiology, National Institute of Mental Health, Klecany, Czech Republic

## Abstract

It is far from conclusive what distinguishes the inattentive (ADD) and the combined (ADHD-C) subtype of ADHD on the neuronal level. Theoretical considerations suggest that especially interval timing processes may dissociate these subtypes from each other. Combining high-density EEG recordings with source localization analyses, we examine whether there are ADHD-subtype specific modulations of neurophysiological processes subserving interval timing in matched groups of ADD (n = 16), ADHD-C (n = 16) and controls (n = 16). Patients with ADD and ADHD-C show deficits in interval timing, which was correlated with the degree of inattention in ADD patients. Compared to healthy controls, patients with ADHD-C display a somewhat weaker, yet consistent response preparation process (contingent negative variation, CNV). In patients with ADD, the early CNV is interrupted, indicating an oscillatory disruption of the interval timing process. This is associated with activations in the supplemental motor areas and the middle frontal gyrus. Patients with ADD display adequate feedback learning mechanisms (feedback-related negativity, FRN), which is not the case in patients with ADHD-C. The results suggest that altered pacemaker-accumulation processes in medial frontal structures distinguish the ADD from the ADHD-C subtype. Particularly in patients with ADD phasic interruptions of preparatory neurophysiological processes are evident, making this a possible diagnostic feature.

## Introduction

Attention Deficit Hyperactivity Disorder (ADHD) is one of the most prevalent neuropsychiatric disorders in childhood^1–3^. Deficits in executive control are common in patients with ADHD^4^. They affect multiple domains of everyday functioning^5–8^ and are likely to emerge from structural abnormalities in medial/superior frontal and basal ganglia structures^9–11^ as well as from dysfunctions in the dopaminergic system^12–14^. However, ADHD is not a homogenous disorder, but patients can be classified as belonging to the inattentive, the hyperactive/impulsive or the combined ADHD subtype depending on symptomatology. The inattentive (ADD) and the combined (ADHD-C) subtype are the most prominent^15,16^. Despite the fact that research has made considerable progress in the understanding of neuronal mechanisms differing between ADD and ADHD-C^17^, the picture showing what dissociates these subtypes is far from clear.

One important cognitive function that has attracted considerable research interest in ADHD refers to ‘time estimation’ and ‘interval timing’^18–25^. These studies examine how well patients with ADHD are able to dissociate different durations of presented stimuli or how well they are able to perform motor actions after a particular time interval has passed (interval timing). Studies on interval timing show dysfunctions in ADHD, which is plausible since dopaminergic mechanisms and frontal-striatal networks - which are affected in ADHD - are central for such processes^18,26,27^.

However, the important and yet unanswered questions are (i) how neurophysiological processes and associated functional neuroanatomical structures are modulated on occasions where the production of time intervals is successful in ADHD and (ii) in how far this makes it possible to distinguish between ADHD subtypes. This is of clinical relevance since it has been suggested that time estimation processes are indeed useful to distinguish between ‘real ADHD’ and ‘pseudo-ADHD’^28^. Conceptual accounts (i.e. pacemaker-counter models)^29,30^ as part of the striatal beat-frequency theory (SBF) explaining timing and time perception processes hold that attention and arousal can speed up or slow down timing by closing or opening a switch that allows pulses generated by an “oscillating clock” to be counted^31^. A faster running “clock” makes time intervals seem longer and it has been suggested that this is the case in ADHD^24^. As both attention and arousal have been suggested to be modulators of the “clock”, patients with different subtypes of ADHD can be expected to show differential interval timing abilities. An attention deficit (as it is common to patients with ADD and ADHD-C) alone would already result in dysfunctions time estimation skills. The hyperactivity/impulsivity occurring solely in patients with ADHD-C significantly increases fluctuations of arousal^32–34^ and thus very likely represents an additional influence on time estimation abilities in this group. Overall, we thus expect for time estimation abilities to vary between the two ADHD-subgroups as the interaction between attention and hyperactivity/impulsivity leads to qualitative differences mainly in arousal.

Neurophysiologically, processes relevant to interval timing are reflected by the ‘contingent negative variation’ (CNV)^35^. The CNV has been shown to be a correlate of interval timing functions as it reflects expectancy^36^ and processes reflected by the CNV are known to be dysfunctional in ADHD^37^. We hypothesize that the pattern of the CNV differs in patients with ADHD compared to controls. Aside from simple amplitude differences, differences in the temporal course of the CNV could occur between the different ADHD subtypes. Especially the early time periods of the CNV immediately following the presentation of a stimulus indicating the beginning of a preparatory/timing processes have been linked to modulations by arousal and attention^38^. Thus, subtype-specific modulations should especially occur in these early phases of interval timing. Since the early CNV involves the supplementary motor area and the anterior cingulate cortex (ACC)^38^ and these areas have been shown to be important for interval timing functions^18,26^, we expect group dependent modulations to be associated with these areas.

However, while theoretical concepts stress the role of attention and arousal in early phases of interval timing, learning mechanisms may also play a very important role^39^. These are usually driven by external feedback to adjust processes subserving interval timing^39,40^; i. e, the theoretical concepts stress that reinforcement learning processes are needed to adjust and select the set of neuronal oscillators that best represent event duration. Such feedback processes are reflected by the feedback-related negativity (FRN). The FRN is affected by the dopaminergic system^41^ and these processes have been shown to be dysfunctional in ADHD^42,43^. It is therefore central to examine whether differences between ADHD-subtypes in interval timing processes may rather be explained by altered integration of external feedback during early phases of interval timing.

## Results

### Behavioural data

The behavioral data, i.e. rate of correct (in-time), early and late responses is given in Table 1. A repeated-measures ANOVA revealed a main effect of *Response* (F(2, 44) = 67.8; p ≤ 0.001; η_p_^2^ = 0.75), showing that significantly more responses were given in the correct time window than too early or too late (both p ≤ 0.001). More errors occurred due to too early than due to too late responses (p = 0.02). We found a significant Response*Group interaction (F(4, 90) = 4.24; p = 0.003; η_p_^2^ = 0.16). Further analysis showed that participants in all three groups displayed more correct than early (all p < 0.04) or late responses (all p < 0.001). In healthy controls and patients with ADD, the number of too early and too late responses did not differ significantly (p > 0.2). Only participants with ADHD-C responded too early more frequently than too late (p = 0.02).

**Table 1:** Occurrence of correct, early and late and missed responses.

	Correct Responses	Early Responses	Late Responses	Misses
Controls	64 ± 12%	17 ± 8%	18 ± 8%	1 ± 0.2%
ADD	44 ± 15%	30 ± 14%	23 ± 10%	3 ± 0.8%
ADHD-C	49 ± 16%	30 ± 15%	18 ± 6%	3 ± 0.7%

Percentage (mean ± standard deviation) of responses in each category and group.

We further examined the relations between behavioural performance on the time estimation task and ADHD symptoms as measured by the ADHD Symptom Checklist^44^. We found a negative correlation (r = −0.54; p = 0.03) between the number of correct responses and the severity of inattention in patients with ADD only. None of the other correlations between accuracy and symptom severity were significant in any of the three groups (all r < 0.4; p > 0.09).

### Neurophysiological data

#### Early visual processing (P1/N1)

**Figure 1:**
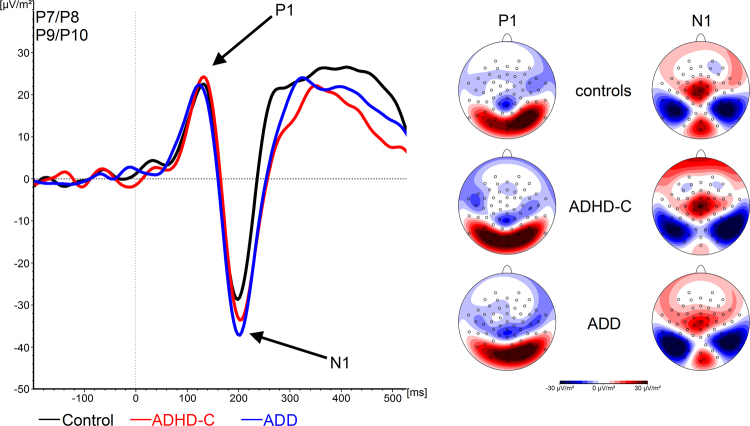
(A) P1 and N1 components occurring in response to the target stimulus in correct trials for healthy controls, patients with ADD and patients with ADHD-C. The scalp topographies show the peak of the amplitudes. Time point zero denotes onset of the stimulus (white square). Negative values are plotted downwards. (B) Corresponding topographical maps, each for every time frame. Positive values are given in red, negative values are given in blue.

The P1 and N1 ERP-components are shown in Fig. 1. Considering the P1 ERP-component, we found no main effects of *Accuracy* (F(1, 45) = 1.05; p = 0.31; η_p_^2^ = 0.02) or *Group* (F(2, 45) = 0.13; p = 0.88; η_p_^2^ = 0.01) and no interaction between these factors (F(2, 45) = 0.21; p = 0.81; η_p_^2^ = 0.01). All analyses containing the factor *Electrode* were also not significant (all F < 1.3; all p > 0.4). Similarly, the repeated-measures ANOVA of the N1 ERP-component also did not reveal a main effect of *Accuracy* (F(1, 45) = 0.03; p = 0.87; η_p_^2^ = 0.01) or *Group* (F(2, 45) = 0.31; p = 0.74; η_p_^2^ = 0.01) or an interaction between them (F(2, 45) = 1.0; p = 0.38; η_p_^2^ = 0.04). All analyses containing the factor *Electrode* were also insignificant (all F < 1.1; all p > 0.6).

### Timing properties (CNV)

**Figure 2:**
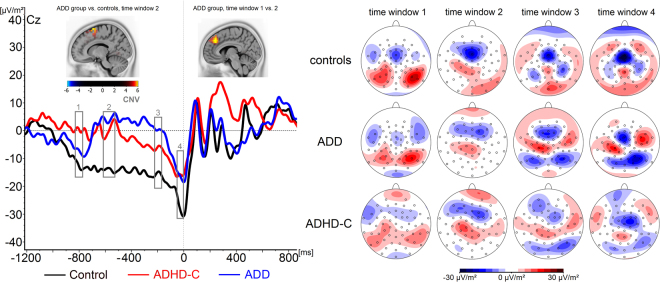
(A) CNV in correct trials for healthy controls, patients with ADD and patients with ADHD-C. Highlighted areas show examined time windows 1–4. Time point zero denotes the time of the given response. Negative values are plotted downwards. S-Loreta images show the source of the amplitude difference between time windows 1 and 2 within the patients with ADD and the differences between healthy controls and the patients with ADD in time window 2. (B) Corresponding topographical maps, one for every time frame. Positive values are given in red, negative values are given in blue.

The CNV is shown in Fig. 2. We found a main effect of *Time Window* (F(3, 43) = 26.9; p ≤ 0.001; η_p_^2^ = 0.65). Pair-wise comparisons revealed significant differences between the four time windows in all cases (all p < 0.02) apart from when comparing time windows 1 and 2 (p = 0.09). This indicates a steady increase of the CNV throughout its course. There also was a main effect of *Group* (F(2, 45) = 6.04; p = 0.005; η_p_^2^ = 0.21), with CNV amplitudes being generally more negative in healthy controls compared to patients with ADD (p = 0.003) and ADHD-C (p = 0.008). The two clinical groups did not differ from each other (p = 0.7). Furthermore, we found a significant interaction between *Time Window*, *Accuracy* and *Group* (F(6, 88) = 2.5; p = 0.026; η_p_^2^ = 0.15). Analyzing this interaction in more detail, it became apparent that there was no *Time Window* * *Group* interaction in error trials (F(6, 88) = 1.4; p = 0.21; η_p_^2^ = 0.09) (see Supplementary Figure 1), but only in trials with on-time responses (correct trials) (F(6, 88) = 2.3; p = 0.04; η_p_^2^ = 0.14). Therefore, the correct trials were analyzed in more detail.

As can be seen in Fig. 2, the ADD group did not reveal a steady increase in CNV amplitude, but showed an oscillatory pattern in the early phase of the CNV. The analysis of the CNV within the ADD group revealed a significant main effect of *Time Window* (F(3, 13) = 7.5; p = 0.004; η_p_^2^ = 0.63). The CNV amplitude turned positive in time window 2 (4.4 ± 14.4 µV/m^2^) compared to time window 1 (−7.3 ± 11.6 µV/m^2^) (p = 0.002), indicating a phasic activity. The sLORETA analysis comparing time window 1 and 2 within the ADD group revealed activation differences in the supplemental motor areas (SMA) (BA6) and the medial frontal gyrus (BA6). Within the ADD group, the CNV amplitude in time window 4 (−16.9 ± 4.2 µV/m^2^) was significantly more negative than that in time windows 2 and 3 (0.03 ± 12.4 µV/m^2^) (both p = 0.001). Such a phasic activity of the CNV (i.e. negative in time window 1 and positive in time window 2) was not seen in ADHD-C patients and controls. Even though there was a main effect of *Time Window* in controls (F(3, 13) = 8.6; p = 0.002; η_p_^2^ = 0.67) and the patients with ADHD-C (F(3, 13) = 9.65; p = 0.001; η_p_^2^ = 0.69), it is shown that both groups were not characterised by a positive-going amplitude change between time windows 1 (controls: −13.3 ± 12.4 µV/m^2^; ADHD-C: −0.06 ± 15.4 µV/m^2^) and 2 (controls: −14.1 ± 17.4 µV/m^2^; ADHD-C: 0.38 ± 14.6 µV/m^2^) (all p > 0.65). In patients with ADHD-C (−17.7 ± 4.2  µV/m^2^) and the control group (−27.2 ± 4.2 µV/m^2^), the CNV was more negative in time window 4 than time window 1 (all p < 0.002). In the ADHD-C group, there were also differences between time windows 1 and 3 (−6.32 ± 14.2 µV/m^2^) as well as between 2 and 3 (all p < 0.05).

Analyzing between-group differences in the different time windows, we found a significant main effect of *Group* (F(2, 45) = 4.03; p = 0.03) in time window 1, showing a significantly more negative early CNV amplitude in healthy controls compared to patients with ADHD-C (p = 0.021) but not when compared to those with ADD (p = 0.61). The two clinical groups did not differ from each other (p = 0.39). In CNV time window 2, the main effect of *Group* was also significant (F(2, 45) = 6.3; p = 0.004): the CNV was significantly more pronounced in healthy controls compared to both patients with ADD (p = 0.005) and ADHD-C (p = 0.034). Again, there were no significant differences between these two patient groups (p > 0.99). The sLORETA analysis revealed differences between the control and the ADD group in the supplemental motor areas (SMA) (BA6) and the medial frontal gyrus (BA6). In CNV time window 3 (main effect *Group*: F(2, 45) = 4.14; p = 0.02), the amplitude in the control group (−15.3 ± 18.2 µV/m^2^) was significantly more negative than that in patients with ADD (p = 0.02), but not when compared to those with ADHD-C (p = 0.3). The difference between the two patient groups was not significant (p = 0.072). No main effect of *Group* was present in CNV time window 4 (F(2, 45) = 1.86; p = 0.17).

#### Feedback processing (FRN)

**Figure 3:**
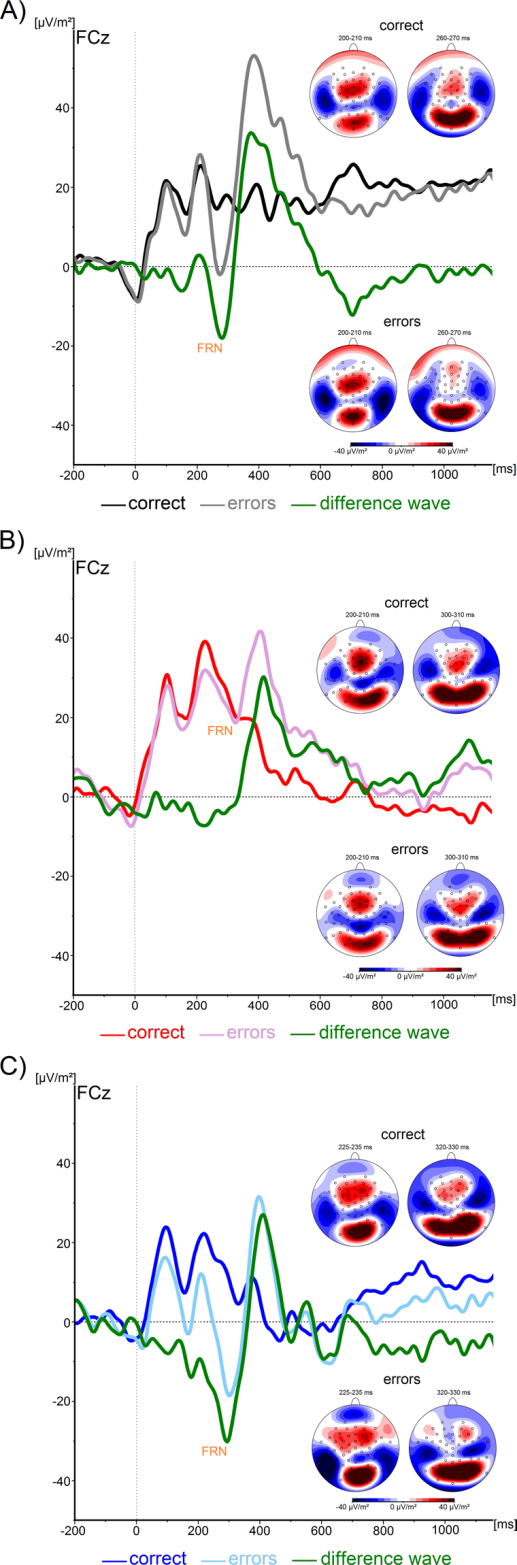
(**A**) FRN shown in correct and error trials in healthy controls. Note the significant negativity in the difference wave, indicating a significantly more pronounced neurophysiological response to negative than to positive feedback. (**B**) FRN shown in correct and error trials in patients with ADHD-C. Note the significant negativity in the difference wave, indicating pronounced neurophysiological response to negative feedback that is similar to that in healthy controls. (**C**) FRN shown in correct and error trials in patients with ADD. Note that no significant negativity is visible in the difference wave, indicating deficit feedback processing in this group compared to healthy controls and patients with ADD.

The feedback-related negativity (FRN) is shown in Fig. 3. For the FRN amplitude, the one-way ANOVA revealed a significant main effect of *Group* (F(2, 45) = 4.8; p = 0.01): the amplitude difference between correct and error trials was larger in healthy controls (−18.2 ± 11.9 µV/m^2^) than in ADHD-C patients (4.9 ± 26.9 µV/m^2^) (p = 0.01). No difference was found between the healthy control group and the ADD patients (−6.8 ± 21.5 µV/m^2^) (p = 0.40). The two clinical groups did not differ from each other (p = 0.37).

## Discussion

In the current study we examined time estimation/interval timing processes in children with ADHD with special emphasis on differential modulation between the ADD and the ADHD-C subtypes. We examined the system neurophysiological processes underlying successful time production processes in these groups. In line with previous studies, the behavioural data show clear time production deficits in ADHD compared to controls^19–21,23,28^. The patients with ADD and ADHD-C performed on a similar level with the only difference being the comparatively low number of late responses in the ADHD-C group. Interestingly, more pronounced symptoms of inattention were associated with fewer correctly timed responses in patients with ADD only. Medication status did not have a significant effect on these results. Importantly, when time production was successful, the neurophysiological data revealed very specific differences between the groups.

These changes were not reflected in basic bottom-up perceptual mechanisms reflected in the P1 and N1 ERP^45–49^, but in the CNV. This suggests that the basic processing of the stimulus indicating to start the timing of the response does not modulate the observed differences between ADHD subtypes. Rather, changes were specific for the timing phase and neurophysiological processes before the timed responses was executed. Most importantly, we observed a multi-phasic pattern in preparatory processes selectively in ADD patients. Initially, the ADD patients and healthy controls showed negative deflections in the CNV that were stronger than in the ADHD-C group (see Table 2).

**Table 2:** Amplitude changes in the CNV across time. Arrows pointing up denote increasing CNV amplitudes, arrows pointing down denote decreasing CNV amplitudes, straight arrows denote no significant CNV changes. Time window 2, in which groups differ the most, is highlighted.

	Time window 1	Time window 2	Time window 3	Time window 4
Healthy controls				
Patients with ADD				
Patients with ADHD-C				

However, thereafter, the ADD group showed a positive deflection about 800 ms prior to the response, which was absent in all other groups. These results suggest that neurophysiological processes supporting time production processes are interrupted in the ADD group. Interestingly, particularly this early period has been suggested to be modulated by arousal and attention^38^ and pacemaker-counter models^29,30^ also hold that attention and arousal modulate timing processes^31^. Neurophysiological evidence shows that these pacemaker processes are reflected by a ramping/climbing of activity^26^ that is likely reflected by the CNV in the EEG^35,38,39^. The ADD group’s main clinical characteristic is a dysfunction in attention/arousal. The data suggest that especially the ADD subgroup is characterized by an imprecise tuning of pacemaker-accumulation mechanisms in that these processes are subject to interruptions. Such interruptions are absent in the ADHD-C group (see Table 2). Considering symptom differences between the two groups, the hyperactivity/impulsivity in ADHD-C patients may have paradoxically increased arousal, thus allowing a more stable and continuous maintenance of the preparatory processes preceding the response^32–34^. The source localization analysis using sLORETA revealed activation differences in the medial frontal cortex including the supplemental motor areas (SMA) (BA6) and the medial frontal gyrus (BA6). These modulations were observed when contrasting the early time window 1 between controls and ADD patients and also when contrasting brain activation within the ADD group between CNV time windows 1 and 2. These regions have frequently been implicated to play a role in interval timing processes^18^. Since these regions have also been shown to reveal structural neuroanatomical changes in ADHD^9–11^, it may be speculated that the observed changes may reflect an indirect effect of an altered structural neuroanatomy in ADHD. Together, it seems that altered pacemaker-accumulation processes in medial frontal structures distinguish between clinically relevant diagnostic categories; i.e. the ADD and the ADHD-C subtype. It seems that the ADD-specific clinical profile, which is mainly characterized by inattention, underlines the observed modulations. Since differences in timing-related neurophysiological processes have repeatedly been demonstrated in patients with ADHD and we were now able to show very specific differences between ADHD subgroups, this aspect may be incorporated in biomarker-^50,51^ and treatment-^52,53^ approaches to ADHD.

Another theoretically important factor, i.e. learning mechanisms driven by external feedback^39^, is unlikely to explain the observed ADHD subtype-specific modulations. Evidence for this comes from the analysis of the FRN. The FRN is affected by the dopaminergic system^41^ and especially dopaminergic deficiencies may reflect the relevant mechanism behind already observed reductions of the FRN in ADHD^42,43^. Importantly, the ADD group did not show reductions in the FRN compared to controls. Such an amplitude reduction was only present in patients with ADHD-C. This shows that there is a double-dissociation between pacemaker-accumulation (interval timing) and feedback processes in ADD and ADHD-C: In patients with ADD, pacemaker-accumulation processes are altered, while feedback-related neurophysiological processes are intact. Patients with ADHD-C display a consistent and stable preparatory period but do not show an adequate neurophysiological reaction to the given feedback. Therefore, interval timing deficits in ADD are likely to be due their profound inattention and changes in error-feedback processing alone are unlikely to explain differences between ADHD subgroups. It seems that even though error feedback information is processed in a similar manner in patients with ADD and controls, this information is not, or cannot, be used by ADD patients to adjust interval neurophysiological timing processes. Patients with ADHD-C, who are in addition characterized by increased activity and impulsivity, are able to successfully initiate and maintain pacemaker accumulation. Albeit speculative, a possible explanation for this could be that this may act as a counter-mechanism to the inattention and leads to a different pattern than it is the case in patients with ADD.

In summary, the study shows that there are ADHD subtype specific modulations in neurophysiological mechanisms subserving interval timing processes in the medial frontal cortex. The results suggest that altered pacemaker-accumulation processes in medial frontal structures distinguish the ADD from the ADHD-C subtype. Only in patients with ADD phasic interruptions of preparatory neurophysiological processes possibly resulting from fluctuations in the tuning to the “clock” are evident, which is in line with theoretical concepts stressing the importance of attentional/arousal processes during interval timing. Such alterations in the tuning of the “clock” are not evident in patients with ADHD-C. Due to the lack of feedback processing, it seems like the “clock” needs to be reset continuously. It is possible that the inherent heightened arousal is what makes adequate performance possible in some cases. Participants’ medication status did not influence these results significantly. This is probably due to the fact that all testings took place in the afternoons when even extended-release medication was no longer effective.

## Materials and Methods

### Sample

In all patients with ADD and ADHD-C, diagnoses had been determined according to established clinical guidelines. This included parent and child interviews, reports from teachers, symptom questionnaires, testing of general IQ level and attention and various medical tests (EEG, audiometry, vision testing, blood test). Patients were only included if they fulfilled diagnostic criteria according to ICD-10 (F90.0, F90.1 or F98.8). Patients were excluded if they presented with additional acute and/or severe psychiatric or somatic comorbidities (e.g. tic disorder, depressive episode, autism). Overall, 16 patients with ADD (11.1 ± 2.0 years, IQ: 96 ± 9.8, 13 male, 7 medicated with immediate or extended release methylphenidate) and 16 patients with ADHD-C (11.8 ± 1.5 years, IQ: 102 ± 11.3, 15 male, 11 medicated with immediate or extended release methylphenidate) were included in the study. Using the ADHD Symptom Checklist^44^ (0: no problems, 3: severe problems), parents rated their children in regards to inattention (ADHD-C: 2.5 ± 0.4, ADD: 1.94 ± 0.4, (t(30) = −3.6, p = 0.001), hyperactivity (ADHD-C: 2.0 ± 0.6, ADD: 0.5 ± 0.4, (t(30) = −8.2, p < 0.001) and impulsivity (ADHD-C: 2.4 ± 0.5, ADD: 0.8 ± 0.6, (t(30) = −7.7; p < 0.001), thus confirming ADHD symptomatology and the two subtypes. A group of healthy control children (n = 16; 11.7 ± 1.2 years, IQ: 99 ± 10.7, 12 male) was included, who did not display symptoms of ADD or ADHD-C according to parental report on the ADHD Symptom Checklist^44^ (inattention:0.4 ± 0.3, hyperactivity: 0.07 ± 0.2, impulsivity: 0.4 ± 0.3). Healthy controls were recruited simultaneously from an in-house data base and via advertisements. None of them had previously participated in a study concerning time estimation. Controls were not included if they presented with acute and/or severe psychiatric or somatic disorders (e.g. tic disorder, depressive episode, autism) according to parent report. Healthy controls and the two patient groups thus did not differ in regards to age (p = 0.85) or IQ (p = 0.32). All subjects and their parents or legal guardians provided written informed consent in accordance with the Helsinki Declaration of 1975, as revised in 2008. The study was approved by the local ethics committee of the Medical Faculty of the TU Dresden.

### Task

**Figure 4:**
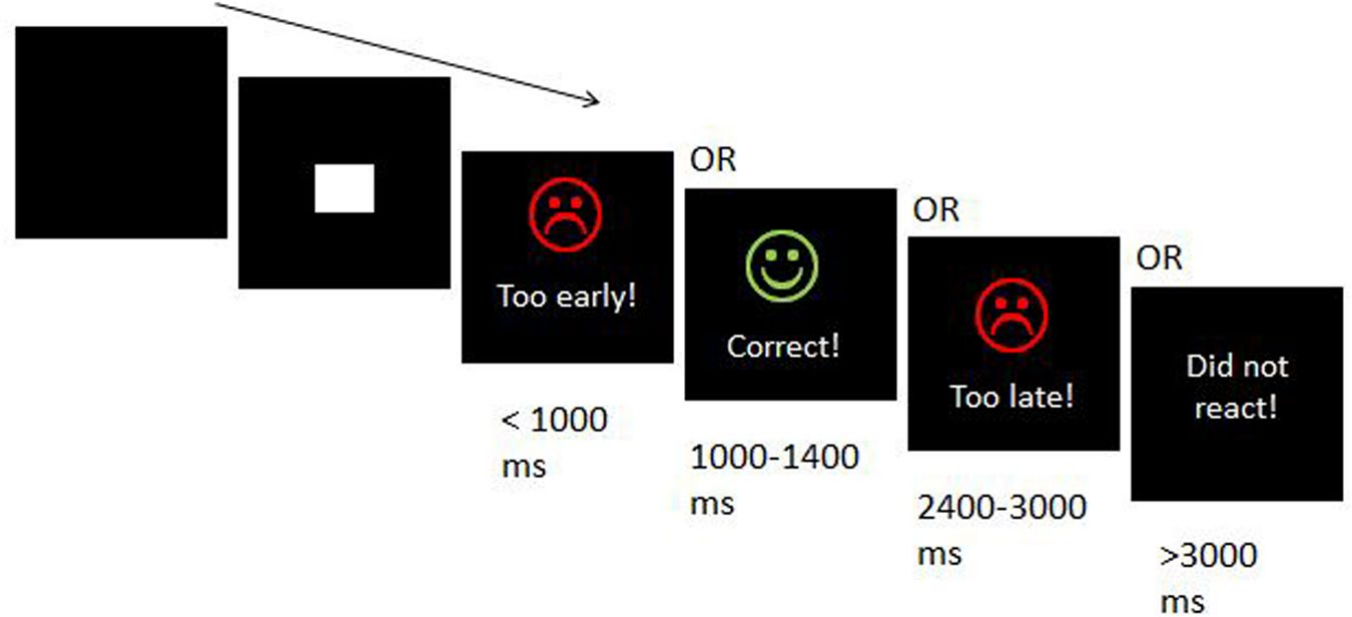
Elements of the used time estimation task. Participants were instructed to estimate 1200 ms from the onset of the stimulus (white square) and to respond with a button press.

The task is shown in Fig. 4.

Participants were asked to estimate a time of 1200 ms following a visual stimulus (white square on black background) and to press a key at the correct time^54,55^. Responses given between 1000 and 1400 ms were accepted as correct. Responses given between 400 and 1000 ms after cue onset were classified as early responses. Responses occurring between 1400 and 2000 ms were classified as late answers. In the neurophysiological analyses, the early and late trials are combined to error trials increase the signal to noise ratio in the analysis. Any key press before 400 ms or after 2000 ms was classified as a missed response and not analyzed further. Visual feedback was given after every key press. In the case of correct trials, a green happy smiley and the word “correct” were presented. In the case of early/late trials (including those later characterized as misses), a red sad smiley and the word “too early”/“too late” was presented. The words “did not react” were displayed if no response had occurred within 3000 ms after stimulus onset. Altogether, participants performed 300 trials in three blocks with short breaks in between them. The intertrial interval was randomized between 800 and 2200 ms. All laboratory testings took place in the afternoon (between 2 and 5 pm) on weekdays.

### EEG recording and analyses

EEG was recorded from 60 equidistant passive Ag/AgCl electrodes (sampling rate: 500 Hz, impedances <5 kΩ, reference electrode at Fpz, ground electrode at θ = 58, ф = 78). Data was pre-processed off-line by down-sampling to 256 Hz. Then, a band-pass filter (0.5–20 Hz, slope of 48 db/oct) was applied and technical artefacts were removed through a raw data inspection. Periodic artefacts (pulse artefacts, horizontal and vertical eye movements) were removed using independent component analysis. This was followed by a segmentation and a baseline correction (see below). Afterwards, an automated artefact rejection procedure was applied to exclude any remaining trials containing artefacts (amplitude criterion: 200 µV/−200 µV; maximal value difference: 200 μV in a 200 ms interval; low activity: below 0.5 μV in a 100 ms period). All data preprocessing procedures combined removed 7 ± 6% of data in the healthy control group, 10 ± 8% in the patients with ADD and 9 ± 7% in the patients with ADHD-C. The proportion of removed segments did not differ significantly between correct and error trials or between the three groups (all p > 0.3). Thus, in all cases a sufficient amount of trials could be included in further analyses. A current source density (CSD) transformation was used to allow a reference-free evaluation of the EEG data and to identify the electrodes showing the strongest effects^56^. At first, data was stimulus-locked and segmented from 2000 ms before to 3000 ms after target onset. The interval from −200–0 ms before the stimulus was used for baseline correction. To examine whether any differences were present between the groups in early visual processing, we examined the P1 (controls: 115–140 ms, patients with ADD and ADHD: 125–150 ms) and N1 (controls: 185–205 ms, patients with ADD and ADHD: 195–215 ms) components over electrodes P7, P8, P9 and P10.

Subsequently, we analysed the preparatory phase before the response (i.e. the contingent negative variation, CNV). To account for the varying time interval between the stimulus and participants’ responses, the stimulus-locked and baseline-corrected data (see above) was now re-segmented and locked to the given response (segmentation window: −2000 ms to 1000 ms around the given response). In this way, we analyzed the processes immediately preceding the response and still maintain a fixed pre-stimulus baseline for all participants. The CNV amplitude was analysed in four separate pre-response time windows in each of the three groups (time window 1: −825–775 ms; time window 2: −625–525 ms; time window 3: −225–175 ms; time window 4: −50–0 ms) at electrode Cz.

Finally, we examined the feedback related negativity (FRN). Here, the unsegmented data was response-locked (segmentation window: −200–1500 ms around the response) and baseline-corrected to the pre-response interval of −200–0 ms. In this way, we analyzed the activity immediately after the response independent of response time variation and independent of the activation occurring before the actual response. To quantify the FRN, we first determined the peak-to-peak amplitude of the two negative deflections (electrode FCz) following the given response in each group (controls & ADD: peak 1: 200–210 ms; peak 2: 300–310 ms; ADHD-C: peak 1: 225–235 ms; peak 2: 320–330 ms). The FRN was then determined as the difference of this amplitude between correct and error trials^57^.

All choices of electrodes and time windows were validated using a statistical procedure described in^58^: Within each of the time windows selected by visual inspection, the mean peak amplitude was extracted for all 60 electrodes. Each electrode was subsequently compared against the average of all other electrodes using Bonferroni-correction for multiple comparisons (critical threshold p = 0.0007). Only electrodes that showed significantly larger mean amplitudes (i.e., negative for N-potentials and positive for the P-potentials) than the remaining electrodes were selected. Of note, this pattern of electrodes matched the electrodes found in the visual inspection of the data. In all neurophysiological analyses, trials with correct responses were contrasted with error trials (average of trials with early and late responses).

Source localisation was conducted using sLORETA (standardized low resolution brain electromagnetic tomography^59^), providing a single solution to the inverse problem^59,60^. For sLORETA, the intracerebral volume is partitioned into 6239 voxels at 5 mm spatial resolution. Then, the standardized current density at each voxel is calculated in a realistic head model^61^ based on the MNI152 template^62^. It has been mathematically proven that sLORETA provides reliable results without a localisation bias^60^. Moreover, there is evidence from EEG/fMRI and neuronavigated EEG/TMS studies underlining the validity of the sources estimated using sLORETA^60,63^. In this study, the voxel-based sLORETA images were compared between groups and between time windows of the CNV within a group using the sLORETA-built-in voxel-wise randomization tests with 2000 permutations, based on statistical nonparametric mapping (SnPM). Voxels with significant differences (p < 0.01, corrected for multiple comparisons) between contrasted conditions were located in the MNI-brain www.unizh.ch/keyinst/NewLORETA/sLORETA/sLORETA.htm.

### Statistical analysis

Mixed effects analyses of variance (ANOVAs) were used to analyze behavioural and neurophysiological data. For the behavioural data, the within-subjects factor *Response* (correct, early, late) and the between-subjects factor *Group* (controls, ADHD-C, ADD) were used for analysis. The analysis of neurophysiological data contained the within-subject factor *Accuracy* (correct, error) and also the between-subject factor *Group* (controls, ADHD-C, ADD). The factor *Electrode* was included whenever necessary (analysis of P1/N1). For the analysis of the CNV, we further used the within-subject factor *Time Window* (time window 1, time window 2, time window 3, time window 4). One-way ANOVAs and t-tests were used to further examine any main effects or interactions. Pearson correlations were used for correlational analyses. When necessary, Bonferroni- and Greenhouse-Geisser-corrections were applied. In all analyses, medication status did not have a significant effect on results (all p > 0.3).

### Data availability

The datasets generated during and/or analysed during the current study are available from the corresponding author on reasonable request.

### Ethical standards

The authors assert that all procedures contributing to this work comply with the ethical standards of the relevant national and institutional committees on human experimentation and with the Helsinki Declaration of 1975, as revised in 2008.

## Electronic supplementary material

Supplementary Figure 1
