# Supplementary material for: Neurophysiological mechanisms of interval timing dissociate inattentive and combined ADHD subtypes
Source: Sci Rep. 2018 Feb 1;8:2033. doi: 10.1038/s41598-018-20484-0 (PMC5794858; doi:10.1038/s41598-018-20484-0)
Supplement: Supplementary file 1 — Supplementary Figure 1 [file 41598_2018_20484_MOESM1_ESM.pdf]

Supplementary Materials

Neurophysiological mechanisms of interval timing dissociate inattentive and combined ADHD subtypes

Annet Bluschke[1,#], Jacqueline Schuster[1], Veit Roessner[1], Christian Beste[1,2]

1 Cognitive Neurophysiology, Department of Child and Adolescent Psychiatry, Faculty of Medicine of the TU Dresden, Germany,
2 Experimental Neurobiology, National Institute of Mental Health, Klecany, Czech Republic

Word count (main text): 4.335

Address for correspondence
Annet Bluschke
Cognitive Neurophysiology, Department of Child and Adolescent Psychiatry, Faculty of Medicine of the TU Dresden, Germany
Schubertstrasse 42, D-01309 Dresden, Germany
Phone: +49-351-458-7072, Fax: +49-351-458-7163
e-mail: annet.bluschke@uniklinikum-dresden.de

Supplementary figure 1:

(A) CNV in error trials for healthy controls, patients with ADD and patients with ADHD-C. Highlighted areas show examined time windows 1-4. Time point zero denotes the time of the given response. Negative values are plotted downwards. (B) Corresponding topographical maps, one for every time frame. Positive values are given in red, negative values are given in blue.

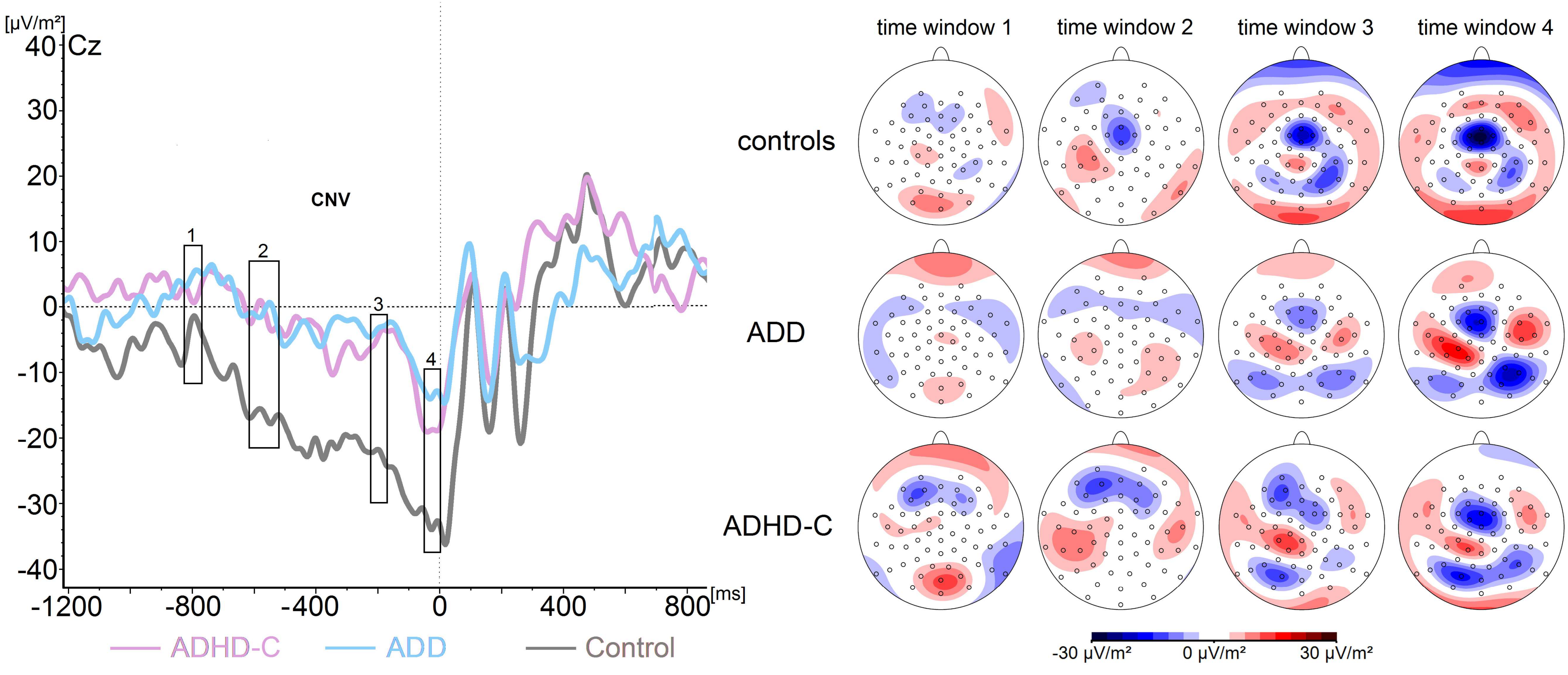

[µV/m²]
Cz
40
30
20
10
0
-10
-20
-30
-40
-1200
-800
-400
0
400
800
[ms]
CNV
1
2
3
4
ADHD-C
ADD
Control
time window 1
time window 2
time window 3
time window 4
controls
ADD
ADHD-C
-30 µV/m²
0 µV/m²
30 µV/m²
